# The Genetic Control of the Compound Leaf Patterning in *Medicago truncatula*

**DOI:** 10.3389/fpls.2021.749989

**Published:** 2022-01-13

**Authors:** Xiaoyu Mo, Liangliang He, Ye Liu, Dongfa Wang, Baolin Zhao, Jianghua Chen

**Affiliations:** ^1^CAS Key Laboratory of Topical Plant Resources and Sustainable Use, CAS Center for Excellence in Molecular Plant Sciences, Xishuangbanna Tropical Botanical Garden, Chinese Academy of Sciences, Kunming, China; ^2^University of Chinese Academy of Sciences, Beijing, China; ^3^School of Life Sciences, University of Science and Technology of China, Hefei, China

**Keywords:** compound leaf development, morphogenesis, pattern formation, leaflet number and arrangement, *Medicago truncatula*

## Abstract

Simple and compound which are the two basic types of leaves are distinguished by the pattern of the distribution of blades on the petiole. Compared to simple leaves comprising a single blade, compound leaves have multiple blade units and exhibit more complex and diverse patterns of organ organization, and the molecular mechanisms underlying their pattern formation are receiving more and more attention in recent years. Studies in model legume *Medicago truncatula* have led to an improved understanding of the genetic control of the compound leaf patterning. This review is an attempt to summarize the current knowledge about the compound leaf morphogenesis of *M. truncatula*, with a focus on the molecular mechanisms involved in pattern formation. It also includes some comparisons of the molecular mechanisms between leaf morphogenesis of different model species and offers useful information for the molecular design of legume crops.

## Introduction

Leaves are the most important photosynthetic organs of plants that produce nutrients, to store, or transport them to other organs. Leaf shape is one of the most diverse morphological features in plant kingdoms, which is the result of the evolutionary adaptation of a species to its specific environment (Warman et al., 2011; Milla, 2012; Schmerler et al., 2012). Two basic types of leaves, simple and compound, are distinguished by the pattern of the distribution of blades on the petiole. Simple leaves have a single undivided blade per petiole. Compound leaves consist of multiple independent blade units, called leaflets, that are attached to the petiole and its derivatives, and organized into specific patterns. Different compound leaves show great variation in a leaf pattern, the leaflet number, and organization, exhibiting much more morphological diversity in nature. Each leaflet is usually considered to be functionally equivalent to a simple leaf, and therefore, the emergence of compound leaves during evolution is thought to have provided some advantages, including increased photosynthetic efficiency and enhanced adaptation to herbivory (Champagne and Sinha, 2004; Warman et al., 2011; Milla, 2012). A major question for plant developmental biologists is the molecular mechanism underlying the diversity of compound leaf forms during evolution.

**FIGURE 1:**
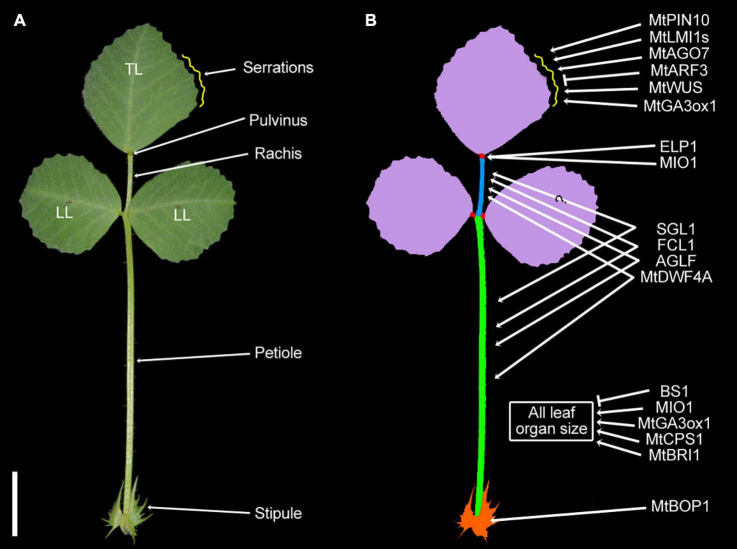
Genes associated with the morphology of the *Medicago truncatula* trifoliate leaf. **(A)** The typical trifoliate leaf of *M. truncatula* consists of a terminal leaflet (TL), a pair of lateral leaflets (LL), a central rachis, and a petiole subtended by a pair of stipules (St). Each leaflet has a pulvinus at the base of the blade, functioning as the motor organ for leaf movement. The distal part of the blade (∼3/4 midvein) forms serrations (yellow curve) along the edges. (Bar, 2 cm.) **(B)** The diagram of the trifoliate leaf and some of the functionally characterized genes involved in regulating the morphology of serrations, pulvinus, rachis, petiole, and rachis of the trifoliate leaf.

In order to study the molecular basis underlying compound leaf development, five model compound-leafed plants are widely used: the Solanaceae tomato (*Solanum lycopersicum*), the Brassicaceae *Cardamine hirsuta*, and three Leguminosae plants *Medicago truncatula*, *Lotus japonicus*, and pea (*Pisum sativum*) (Wang and Chen, 2013; Bar and Ori, 2015). Several excellent related reviews have previously been published, but these are either relatively old or focus primarily on the species tomato and *C. hirsuta* rather than *M. truncatula* and Leguminosae plants (Hofer and Ellis, 1998; Goliber et al., 1999; Kessler and Sinha, 2004; Blein et al., 2010; Efroni et al., 2010; Koenig and Sinha, 2010; Bar and Ori, 2014; Du et al., 2018; Wang H. et al., 2021). The leaves of Leguminosae plants, however, display a great diversity in compound leaf patterns, ranging from pinnate and palmate to higher-ordered complicated, and leaflets in these leaves usually show almost uniform morphology (He et al., 2020); moreover, some species from the Cercideae tribe (Caesalpinioideae) and the Desmodium genus (Papilionoideae) have a simple leaf morphology (Sprent, 2007, 2008). Over the last two decades or so, the molecular basis of this morphological diversity in Leguminosae plants has attracted a high level of interest (Hofer et al., 1997, 2009; Hofer and Ellis, 1998; Champagne et al., 2007; Wang et al., 2008, 2013; Hofer and Noel Ellis, 2014; Jeong et al., 2017; Moreau et al., 2018; Jiao K. et al., 2019). Recent studies in model legume *M. truncatula* identified several regulators involved in the compound leaf development, leading to a growing knowledge of the genetic control of the compound leaf patterning (Chen, 2018; Table 1; Figures 1A,B). *M. truncatula* is a close relative of alfalfa (*Medicago sativa*), the most cultivated forage plant that represents the most economically valuable forage for animal feed but has a limited genetic base for breeding programmers (Chen et al., 2020; Shen et al., 2020). This review aims to summarize the current knowledge about how the leaf morphogenesis of *M. truncatula* is regulated by the coordination of genetic factors, hormones, and other signals, and finally to pattern the compound leaf. It also includes some comparisons of the molecular mechanisms between leaf development of different model species and offers useful information for the molecular design of legume crops.

**TABLE 1:** Functionally characterized genes involved in compound leaf development.

Genes	Annotation of the encoded proteins	Function in leaf development	References
*SINGLE LEAFLET1* (*SGL1*)	FLORICAULA (FLO)/LEAFY (LFY) ortholog	Lateral leaflet initiation; petiole length	Wang et al., 2008
*PALMATE-LIKE PENTAFOLIATA1* (*PALM1*)	Cys(2)His(2) zinc finger transcription factor	Leaflet number and arrangement	Chen et al., 2010; Peng et al., 2017
*SMOOTH LEAF MARGIN1* (*SLM1*)/*MtPIN10*	An auxin efflux carrier protein homologous to *Arabidopsis* PIN-FORMED1 (PIN1)	Terminal leaflet number; lateral leaflet number; marginal serrations	Peng et al., 2011; Zhou et al., 2011
*Fused Compound Leaf1* (*FCL1*)	A class M KNOX protein homologous to *Arabidopsis* KNATM	Boundary formation between leaflets; petiole and rachis length	Peng et al., 2011
*STENOFOLIA* (*STF*)	WUSCHEL-like homeobox (WOX) transcriptional regulator	Blade expansion in the mediolateral axis; leaf vascular patterning	Tadege et al., 2011; Zhang et al., 2014
*MtNAM/MtCUC2*	CUC/NAM transcription factor	Boundary formation between leaflets	Cheng et al., 2012
*NODULE ROOT* (*NOOT*)/*MtBOP1*	A BTB/POZ-ankyrin domain protein orthologous to *Arabidopsis* BOPs	Stipule	Couzigou et al., 2012
*ELONGATED PETIOLULE1* (*ELP1*)/*PETIOLULE-LIKE PULVINUS* (*PLP*)	A LOB DOMAIN-CONTAINING PROTEIN (LBD) transcription factor homologous to *Arabidopsis* LOB	Pulvinus	Chen et al., 2012; Zhou et al., 2012
*MtAGO7*/*LOBED LEAFLET1* (*LOL1*)	An ortholog of *Arabidopsis* ARGONAUTE7 (AGO7)	Marginal serrations	Zhou et al., 2013; Peng et al., 2017
*MtPHAN*	ARP MYB transcription factor	Leaf adaxial–abaxial polarity; blade planar shape; lateral leaflet placement; stipule; marginal serrations	Ge et al., 2014; Zhou et al., 2014
*BIG SEEDS1* (*BS1*)	A TIFY transcription factor homologous to Arabidopsis PEAPOD1 (PPD1) and PPD2	Leaf organ size	Ge et al., 2016
*MtBRI1*	A leucine rich repeat receptor protein kinase (LRR-RLK)	Leaf polarity; blade planar shape; leaf organ size	Cheng et al., 2017; Kong et al., 2021
*HEADLESS* (*HDL*)/*MtWUS*	WUSCHEL homolog	Proximal–distal growth; marginal serrations	Meng et al., 2019; Wang et al., 2019
*AGAMOUS-LIKE FLOWER* (*AGLF*)/*AGAMOUS AND TERMINAL FLOWER* (*AGTFL*)	A nucleus-localized protein containing a putative Myb/SANT-like DNA-binding domain and a PKc kinase domain	Proximal–distal growth; petiole and rachis length	Zhang et al., 2019; Zhao et al., 2019; Zhu et al., 2019
*PINNATE PENTAFOLIATA1* (*PPF1*)/*MtREV1*	Class III homeodomain-leucine zipper (HD-ZIPIII) transcription factor	Leaflet number and arrangement; adaxial–abaxial polarity of terminal leaflet	Zhou et al., 2019
*Lateral Leaflet Suppression 1* (*LLS1*)/*MtYUCCA1*	A flavin monooxygenase homologous to *Arabidopsis* YUCCA1	Outgrowth of lateral leaflet; leaf venation	Zhao et al., 2020
*PINNATE-LIKE PENTAFOLIATA1* (*PINNA1*)	A BEL1-like homeodomain protein homologous to *Arabidopsis* BLH11	Leaflet number and arrangement	He et al., 2020
*Dwarf and Increased Branching 1* (*DIB1*)/*SMALL AND SERRATED LEAF* (*SSL*)/*MtGA3ox1*	*Arabidopsis* GA3-oxidase 1 (GA3ox1) homolog	Leaf organ size; petiole and rachis length; marginal serrations	Zhang et al., 2020; Wen et al., 2021
*Mini Plant 1* (*MNP1*)/*MtCPS*	Copalyl diphosphate synthase	Leaf organ size; petiole and rachis length	Guo et al., 2020
*MINI ORGAN1* (*MIO1*)*/SMALL LEAF AND BUSHY1* (*SLB1*)	F-box protein	Leaf organ size; proximal–distal growth; pulvinus	Yin et al., 2020; Zhou et al., 2021
*WRINKLED FLOWER AND LEAF* (*WFL*)	3-ketoacyl-CoA synthase	Cuticular wax; leaflet separation; blade planar shape	Yang et al., 2021
*MtDWARF4A* (*MtDWF4A*)	A cytochrome P450 protein orthologous to *Arabidopsis* DWARF4	Length of petiole, rachis, and pulvinus; blade planar shape	Kong et al., 2021; Zhao et al., 2021
*MtLMI1a* and *MtLMI1b*	HD-Zip I transcription factors homologous to *Arabidopsis* LMI1	Marginal serrations	Kong et al., 2021
*MAIN STEM DWARF1* (*MSD1*)/*MtGA20ox1*, *MtGA20ox7*, and *MtGA20ox8*	GA 20-oxidase	Leaf organ size	Li et al., 2021

## Pattern Formation and Morphogenesis in Compound Leaf Development of *Medicago truncatula*

The ontogeny of leaf development can be conventionally divided into three continuous and overlapping phases: initiation, primary morphogenesis, and secondary morphogenesis (Efroni et al., 2010; Bar and Ori, 2015). Leaf primordia that lead to either simple or compound shapes are initiated from the flanks of the shoot apical meristem (SAM) and produced in series separated by a time period termed plastochron (P). Universally, leaf primordia at sequential orders of plastochrons (P1, P2, P3, and P4…) are used to describe their developmental stages. The latest emerging leaf primordium is termed P1, the next oldest leaf primordium P2, and so forth, whereas the leaf founder cell population is designated as P0. Leaf initiation, that is stages P0 to P1, refers to the process of the recruitment of leaf founder cells (P0) on the peripheral zone of SAM and the subsequent formation of a protrusion (P1) after early cell division. After initiation, leaf primordia proceed with the second phase, the primary morphogenesis, which includes the establishment of three polarity axes (adaxial–abaxial, proximal–distal, and mediolateral), the specification of the primordial lamina, petiole, and other organs in leaves (i.e., leaflets in compound leaves) and the formation of marginal structures such as lobes and serrations (Du et al., 2018). The final phase is secondary morphogenesis, which involves limited cell division and extensive cell expansion and differentiation, leading to the attainment of final leaf shape and size (Gonzalez et al., 2012). Because of the main role of secondary morphogenesis in promoting laminar outgrowth and organ elongation rather than organ pattern of the leaf, it is generally believed that the controlling mechanisms of this process are likely to have greater similarities than differences between simple and compound leaf development. Overall, different from simple leaf development, compound leaf development contains a specific morphogenetic process during the primary morphogenesis, namely the formation of separated leaflet primordia, and this process largely determines the final leaflet number and arrangement (He et al., 2020). Therefore, in this review, the authors mainly dissect the regulators and pathways controlling the primary morphogenesis of the compound leaf in *M. truncatula*, with a particular concern about the mechanisms responsible for the pattern formation.

In *M. truncatula*, the first leaf following the appearance of the cotyledons is unifoliate in the juvenile form in contrast to subsequent trifoliate leaves in adult form, which consist of a pair of lateral leaflets and a terminal leaflet at the distal end of a petiole subtended by a pair of stipules (Wang et al., 2008; Figure 1A). Each leaflet in either unifoliate leaves or trifoliate leaves has an independent pulvinus at the base of the lamina, functioning as the motor organ for the nyctinastic leaf movement (Chen et al., 2012; Zhao et al., 2021). Our description and discussion are concentrated on the development of the trifoliate leaves where most studies have been focused on.

**FIGURE 2:**
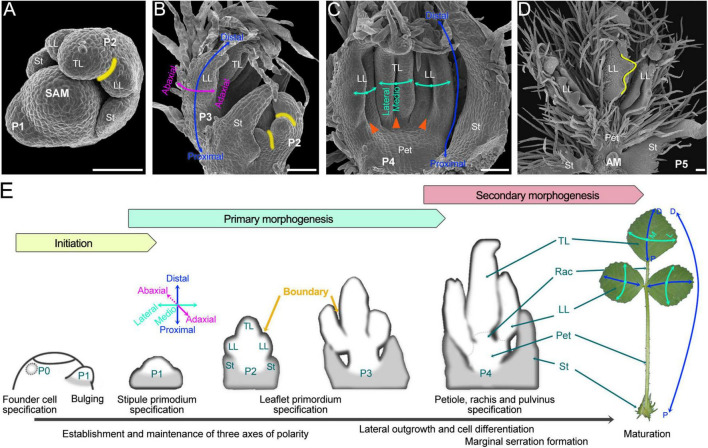
The ontogeny of compound leaf development in *M. truncatula*. **(A–D)** SEM images of the shoot apical meristem (SAM) and/or developing leaf primordia. **(A)** The organization of the shoot apex with two leaf primordia developed at the SAM periphery. Primordia are named according to the plastochron (P) age: the latest emerging primordium is termed P1, the next oldest primordium P2, etc. The yellow curve marks the boundary between the terminal leaflet (TL) and the lateral leaflet (LL) primordia. St, stipule. **(B)** The organization of the shoot apex shows the SAM protected by P2 and P3 leaf primordia. Curved arrows mark the adaxial–abaxial (pink) and proximal–distal (blue) axes of leaf asymmetry. **(C)** Adaxial side view of the P4 leaf primordium. Cyan curved arrows mark the mediolateral axis. During stages P3 to P4, due to the abaxial surface outgrows the adaxial surface, the leaflet primordia became folded (orange triangle). **(D)** Adaxial side view of the P5 leaf primordium with the serrations (yellow curve) being formed. Pet, petiole. (Bars, 50 μm). **(E)** Diagrams of compound leaf primordia at successive stages of ontogeny. The leaf development of *M. truncatula* can be divided into three successive phases. The first is the initiation of leaf primordium from the peripheral zone of SAM. The following is primary morphogenesis, during which three axes of leaf polarity are established, and three separated leaflet primordia, as well as primordial petiole and rachis, are formed. The last is secondary morphogenesis, during which the vasculature, leaf margins, and other specialized epidermal cells such as trichomes and stoma accomplish their differentiation to make a mature leaf. A mature trifoliate leaf exhibits both global and local polarity along the proximal-distal axis (rightmost): the global proximal-distal polarity is manifest in the distribution of distinct specialized organs along the proximal-distal axis (blue and long double-headed arrow), while each leaflet exhibits independent local proximal-distal polarity (blue and short double-headed arrows). Rac, rachis.

The trifoliate leaf is initiated as a strip of cells termed common primordia (P1) outgrowth along the SAM periphery, and later it progresses into primary morphogenesis, which includes most critical developmental events determining the leaf pattern (Wang et al., 2008; Chen, 2019; Figure 2). At the late P1 stage, stipule primordia become apparent at the proximal end of the common leaf primordium. During the P2 stage, a pair of lateral leaflet primordia were initiated between the stipule and the common primordium which later was differentiated into the terminal leaflet primordium; at the same time, boundaries were sequentially established between the stipule and lateral leaflet primordia, and between the lateral and terminal leaflet primordia (Figure 2A). Up to the late P3 stage, the three separated leaflet primordia were already formed, and the basic structure of the trifoliate leaf was established (Figure 2B). Then, during stages P3 to P4, another important and characteristic developmental event is that the abaxial surface of each leaflet primordium outgrows the adaxial surface, resulting in the leaflet primordia becoming folded (Figure 2C). Beginning from the P4 stage, the specification of petiole, rachis, and pulvinus and the formation of marginal serrations occurred in succession (Figures 2D,E). Primary morphogenesis occurs during very young stages when the leaf is still protected by older leaves at the shoot apex, while secondary morphogenesis encompasses a much longer time period and represents an increase in surface area and volume of several thousandfold. Trichomes, as a maker of cell differentiation, firstly emerge in late P2 from the abaxial surface of the terminal leaflet primordium and later were gradually developed from the stipule, lateral leaflet, petiole, and rachis primordia, and up to P5 stage, they densely covered the leaf surfaces, with especially abundance on the abaxial surface and the petiole and rachis (Figures 2A–D). During P5 and later stages, leaf cells mainly undergo cell-fate determination, differentiation, and expansion; the vasculature, leaf margins, and other specialized epidermal cells such as trichomes and stoma accomplish their differentiation to make a mature leaf. Usually, up to the P8 stage, the flattened shape of leaves was established.

## Leaf Initiation

The SAM is the source of all cells that ultimately form the above-ground architecture of plants, including the subset that ends up building the leaves (Vernoux et al., 2021). The homeobox gene *WUSCHEL* (*WUS*), which is specifically expressed in the organizing center of the SAM, plays a central role in the formation and maintenance of the shoot meristem activity (Schoof et al., 2000); loss-of-function mutations in *WUS* result in the misspecification of stem cells and the premature termination of meristem activity (Lenhard et al., 2001). In *M. truncatula*, in addition to a conserved role in the SAM and axillary meristem maintenance, the *HEADLESS* (*HDL*)/*MtWUS* has been implicated in the regulation of both the leaf proximal–distal axis elongation and the leaf margin morphology (Meng et al., 2019; Wang et al., 2019).

Leaf founder cells are part of the SAM and cannot be easily distinguished from other cells in histological appearance, but have certain cellular characteristics, including dense cytoplasm, very small vacuoles, and a high cell division rate (Verdeil et al., 2007; Yruela, 2015). The acquisition of founder cell identity is determined by specific gene expression and hormone signaling programs, and some molecular characteristics are: (i) the class I *KNOTTED1-LIKE HOMEOBOX* (*KNOXI*) genes that principally function to maintain the SAM identity are excluded from founder cells (Long et al., 1996; Hake et al., 2004); (ii) genes promoting organ specification and differentiation are activated, such as *ASYMMETRIC LEAVES1*/*ROUGH SHEATH2*/*PHANTASTICA* (collectively named *ARP*) and other adaxial–abaxial polarity genes (Fukushima and Hasebe, 2014); (iii) PIN-mediated the local auxin accumulation triggers the primordium bulging (Furutani et al., 2014). The initiation program would be highly conserved in *M. truncatula* which was evident from the following two considerations. At first, *KNOXI* genes are down-regulated at the site of the incipient leaf primordium where some adaxial–abaxial polarity genes such as *M. truncatula ARGONAUTE7* (*MtAGO7*)/*LOBED LEAFLET1* (*LOL1*), *AUXIN RESPONSE FACTOR3* (*MtARF3*), *PINNATE PENTAFOLIATA1* (*PPF1*)/*MtREVOLUTA1* (*MtREV1*), and *MtYABBY3* are activated (Zhou et al., 2013, 2014; Peng et al., 2017). Secondly, the initiation is proved to correspond to transient auxin maxima that probably generated by both *Lateral Leaflet Suppression 1* (*LLS1*)/*MtYUCCA1*-mediated local biosynthesis (Zhao et al., 2020) and *SMOOTH LEAF MARGIN1* (*SLM1*)/*MtPIN10*-mediated polar transport (Peng and Chen, 2011; Zhou et al., 2011). Their detailed descriptions have been discussed the below sections.

## LFY Plays a Central Role in the Trifoliate Pattern Formation

How ordered leaflets are formed to a specific pattern is a fundamental question of compound leaf development. The pattern formation is largely dependent on maintaining and modulating a transient morphogenetic activity in the early leaf primordia which directs temporal and spatial patterns of the leaflet initiation (Bar and Ori, 2015). In most compound-leafed species including tomato and *C. hirsuta*, in addition to the role in promoting and maintaining SAM indeterminacy, *KNOX I* genes also play a key role in maintaining transient indeterminacy and morphogenetic activity in early compound leaf primordia (Hareven et al., 1996; Hay and Tsiantis, 2006; Challa et al., 2021). As mentioned above, *KNOX I* genes are specifically expressed in SAM and are down regulated in the incipience of leaf primordium across angiosperms (Vollbrecht et al., 1991; Bharathan et al., 2002). The down-regulation of *KNOX I* genes expression is permanent during simple leaf development, but short-term expression activation occurs in the early stages of compound leaf development, resulting in the initiation of leaflet primordia from the margin of compound leaf primordia (Hake et al., 2004; Hay and Tsiantis, 2006). Overexpression of *KNOX I* genes led to extra leaflet production (Hareven et al., 1996; Hay and Tsiantis, 2006), while the loss-of-function mutation in *KNOX I genes* resulted in simple-like leaves (Rast-Somssich et al., 2015). The reactivation of *KNOX I* genes in developing compound leaves is also seen in most legumes, but not detected in some legumes belonging to the inverted-repeat-lacking clade (IRLC), including pea and *M. truncatula* (Champagne et al., 2007). In IRLC legumes, the role of *KNOX I* genes is completely replaced by the *FLORICAULA* (*FLO*)/*LEAFY* (*LFY*) orthologs *UNIFOLIATA* (*UNI*) and *SINGLE LEAFLET1* (*SGL1*) (Hofer et al., 1997; Wang et al., 2008; Zhou et al., 2014). *LFY* and its orthologs are highly conserved in plants and play a central role in specifying floral meristem identity in angiosperms (Sayou et al., 2014). In both pea and *M. truncatula*, loss-of-function mutation of *SGL1* not only leads to defective in floral development, producing inflorescence-like structures but also converts compound leaves into simple-like leaves (Hofer et al., 1997; Wang et al., 2008). *M. truncatula SGL1* has specifically expressed in central regions of the earliest leaf common primordia, later in terminal leaflet primordia, and last in the developing rachis (He et al., 2020; Zhao et al., 2020); this expression pattern is remarkably similar to that of *ChSTM* in the earliest stages of leaf development in *C. hirsuta* (Barkoulas et al., 2008). Interestingly, in some non-IRLC legumes, that is, soybean (*Glycine max*), *L. japonicus*, and mungbean (*Vigna radiata*), RNAi silencing or loss-of-function mutations of *LFY* orthologs also resulted in decreased leaflet number or even a simple leaf-like pattern, despite that *KNOX I* genes are detected in the leaf primordia (Champagne et al., 2007; Wang et al., 2013; Jiao K. et al., 2019), suggesting important roles for both *KNOX I* and *LFY* genes in leaf pattern formation in these species. It is not clear at present how *KNOX I* and *LFY* are coordinated to orchestrate the compound leaf development of the non-IRLC legumes.

During compound leaf pattern formation, how the KNOX I or LFY/SGL1-associated morphogenetic activity is directly regulated to ensure a correct leaf pattern is a central question. Recent publications suggest that the class II KNOX genes (*KNOX II*) (*KNAT3*, *KNAT4*, and *KNAT5*) confer opposing activities with KNOX I genes to suppress leaflet initiation in the simple leaf developmental program of Arabidopsis (Furumizu et al., 2015; Challa et al., 2021). Two important transcription factors were reported as repressors of the *SGL1* expression during the pattern formation of the *M. truncatula* trifoliate leaves. *PALMATE-LIKE PENTAFOLIATA1* (*PALM1*) encodes a Cys(2)His(2) zinc finger transcription factor that directly binds to the *SGL1* promoter region and represses its transcription (Chen et al., 2010). *PALM1* especially acts in the lateral leaflet primordia; in *palm1* mutants, *SGL1* expression was ectopically detected in the lateral leaflet region that caused two (rather than one) pairs of lateral leaflet formation, resulting in five leaflets organized into a palmate-like pattern. *PINNA1-LIKE PENTAFOLIATA1* (*PINNA1*) gene encodes a BEL1-like homeodomain protein and its loss-of-function mutations led to a compound leaf pattern of five leaflets arranged pinnately (He et al., 2020; Wang and Jiao, 2020). PINNA1 proteins also directly bind to specific regions of the *SGL1* promoter and inhibit the transcription. *PINNA1* expression was found in both terminal and lateral leaflet primordia, and predominantly in the adaxial domain of the leaflet. In *pinna1* mutants, an ectopic *SGL1* expression was observed only in the early terminal leaflet primordia that thus initiated two additional leaflet primordia at the base, but not in the lateral leaflet primordia because of the existence of the functional *PALM1* genes.

**FIGURE 3:**
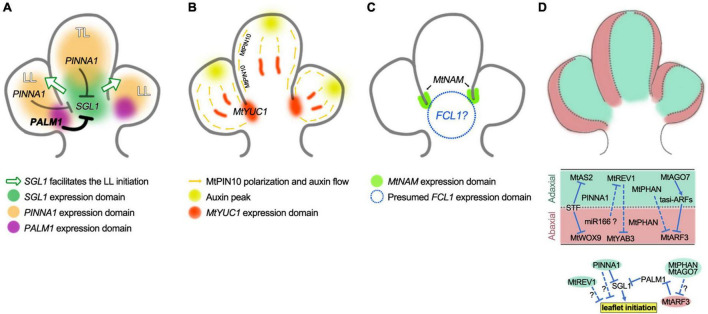
Genes-network controlling the trifoliate pattern formation of *M. truncatula*. **(A)** Model for LFY/SGL1 pathway in trifoliate pattern formation with the diagram representing the P3 leaf primordium of three leaflet primordia but without stipule primordia. *SGL1* is the key indeterminacy factor that maintains a transient morphogenetic activity and promotes the initiation of LL primordia (green arrows), while both *PINNA1* and *PALM1* negatively regulate the morphogenetic activity by directly inhibiting the expression of *SGL1*. *PINNA1* acts alone in the TL primordia and plays a secondary role in the LL primordia (gray lines), where *PALM1* functions as a master role (bold line). **(B)** Model for auxin actions during the trifoliate pattern formation. *MtPIN10* polarization mediates auxin transport leads to the formation of auxin peaks to induce both TL and LL primordia bulging, and the *MtYUC1* encoding auxin biosynthetic enzyme plays an essential role in LL growth. **(C)** Regulation of boundary development. *MtNAM* facilitates the separation of TL and LL primordia by inhibiting cell proliferation in the boundary region, *FCL1* also plays a key role in the development of boundaries between TL and LL primordia. **(D)** Schematic view of the adaxial–abaxial patterns in leaflet primordia of the P3 leaf (upper panel), regulatory networks for the establishment and maintenance of adaxial–abaxial polarity (middle panel), and links between adaxial–abaxial polarity genes and the leaflet initiation (lower panel). *MtAS2*, *MtAGO7*, *MtREV1*, and *PINNA1* are expressed in the adaxial domain of leaf primordia, *MtWOX9*, *MtARF3*, and *MtYAB3* are expressed in the abaxial domain, and *MtPHAN* is expressed in both of the adaxial and abaxial domain, while *STF* is expressed at the adaxial–abaxial junction (middle domain). *STF* directly represses the expression of *MtAS2* and *MtWOX9*, while *MtPHAN* and *MtAGO7* negatively regulate transcription of *MtARF3*, *MtREV1* represses the expression of *MtYAB3*. The adaxial–abaxial polarity genes *MtPHAN*, *MtAGO7*, and *MtARF3* regulate the leaflet initiation through the LFY/SGL1 pathway. The adaxial polarity gene *MtREV1* regulates the leaflet initiation through unknown mechanisms. The *PINNA1* is mainly expressed in the adaxial domain and it negatively regulates the leaflet initiation through both the LFY/SGL1 pathway and an unknown mechanism independent of the *SGL1* gene.

The combination of *palm1* and *pinna1* mutations results in higher-ordered compound leaves consisting of two orders and up to 13 leaflets (He et al., 2020). The five first-order leaflets in a palmate arrangement are formed in a process identical to that described for the five leaflets in *palm1* leaves, while the second-order leaflets located on the petiolules of the first-order leaflets are formed in a manner similar to the additional pair of leaflets developing in *pinna1* leaves. Elevated *SGL1* expression was found to be associated with all leaflet production. The *in vivo* and *in vitro* biochemical analysis revealed that both PALM1 and PINNA1 proteins can bind to the *SGL1* promoter to repress its expression, and they also can form a protein complex. In conclusion, during the pattern formation of trifoliate leaves, PINNA1 acts alone in the terminal leaflet region, while PALM1 functions as a “master regulator” role and PINNA1 as a “secondary regulator” role, to repress the *SGL1* expression and the associated morphogenetic activity (Figure 3A). In another word, either PINNA1 or PALM1 could repress the *SGL1* in the terminal or lateral leaflets region respectively, but PINNA1 only repress *SGL1* in the absence of PALM1.

## Auxin Is Important for Leaflet Initiation and the Subsequent Outgrowth

The compound leaf development in different species is tightly associated with several hormones signaling pathways. Among them, auxin received particular attention that numerous auxin biosynthetic enzymes, transporters, and signaling components have been reported to be involved in multiple processes of leaf development, including leaf and leaflet initiation, leaflet separation, and patterning, and blade outgrowth (Xiong and Jiao, 2019). Auxin is essential for the initiation of leaf common primordia from the flanks of SAM and leaflet primordia from the margins of leaf common primordia. Accumulation of auxin in initiating organs is driven by the activity of the PIN family of auxin efflux carriers (Wisniewska et al., 2006). In *C. hirsuta*, loss-of-function of *PINFOR MED1* (*ChPIN1*) resulted in a decreased leaf production and a simple-like leaf pattern (Barkoulas et al., 2008). The ChPIN1 protein establishes local auxin activity maxima at leaf margin in the same manner as that it functions in SAM periphery, and thus facilitates the formation of lateral leaflets. In *M. truncatula*, SMOOTH LEAF MARGIN1 (SLM1)/MtPIN10 is a functional auxin efflux transporter orthologous to PIN1 (Peng and Chen, 2011; Zhou et al., 2011). The compound leaf development in *slm1*/*mtpin10* mutants exhibited severe defects, including the decrease in marginal serrations, increase in terminal leaflet number, and a simultaneous reduction in lateral leaflet number, accompanied by reduced expression of *SGL1* (Zhou et al., 2011). MtPIN10 is apically localized at the epidermal cells of the leaf primordia and marks the site of incipient primordia, and it also directs auxin maxima at the tips of serrations. It is interesting that the defects in the compound leaf pattern of *slm1/mtpin10* (not much change in leaflet number) are significantly different from that of *chpin1* (almost no leaflet), indicating the auxin regulators acting in diverse manners in different developmental contexts.

Auxin biosynthetic enzymes like YUCCAs are also reported to be necessary for the initiation of leaf and leaflet primordia and the blade outgrowth. A conserved function of YUCCAs on leaf vascular development was found in several species. The loss-of-function of multiple *YUCCA* genes in Arabidopsis resulted in plants with auxin-deficient phenotypes of short and narrow leaf blades and reduced leaf veins (Zhao et al., 2001; Cheng et al., 2007). The *yucca1* mutant of pea (*crispoid*/*psyuc1*) has altered vein density and placement, with missing or underdeveloped tendrils and leaflets at low frequency (McAdam et al., 2017). In *M. truncatula*, the *Lateral Leaflet Suppression1* (*LLS1*) gene encodes a key auxin biosynthetic enzyme MtYUCCA1, which plays a very important role in the compound leaf pattern formation (Zhao et al., 2020). The *lls1* mutants are sever defective in vascular tissue development of blade, and the outgrowth of lateral leaflets was significantly suppressed with a morphology ranging from severely malformed or underdeveloped to small blade size. *MtYUCCA1* is expressed in the emerging primordia as early as P0 and P1 stages, then at the basal regions between the terminal leaflet and lateral leaflet primordia of P2 and P3 stages, and the vascular tissues of leaflet primordia at later stages. As YUCCA proteins convert indole-3-pyruvic acid IPA to natural auxin IAA (Cao et al., 2019), the development of leaflets in trifoliate leaves therefore should be dependent on MtYUCCA1-catalyzed auxin generation and MtPIN10-driven auxin redistribution (Figure 3B).

How auxin signals are translated into programs of compound leaf morphogenesis remains to be investigated. In the most-studied auxin-signaling pathway, class A AUXIN RESPONSE TRANSCRIPTION FACTORS (ARFs) bind to target genes to activate downstream gene expression, whereas Aux/IAA proteins inhibit the auxin response by interacting with ARF activators to inhibit gene expression (Xiong and Jiao, 2019; Israeli et al., 2020). In tomato, multiple ARFs were found to stabilize the developmental output of auxin during leaf patterning, wherein the class A ARFs, SlMP, SlARF19A, and SlARF19B function to promote leaflet initiation and outgrowth but are repressed in the intercalary regions between leaflets by the Aux/IAA protein ENTIRE (E) (Israeli et al., 2019). In *M. truncatula*, overexpression of the *MtARF3* leads to a curling leaf margin with deep serrations or even palmate-like pentafoliate leaves with radialized blades in some cases, and MtARF3 proteins can directly interact with the PALM1 promoter to negatively regulate *PALM1* expression (Zhou et al., 2013; Peng et al., 2017; Figure 3D). The role of MtARF3 in specifying abaxial cell fate is further discussed in the below section “Roles of the leaf polarity genes in the leaf pattern formation” but understanding the detailed molecular mechanism of MtARF3 in the auxin signaling pathway requires further research.

## Boundary Development During Compound Leaf Pattern Formation

Compound leaf development is characterized by the formation of separated leaflet primordia during the early stages of ontogeny. Leaflet primordia are initiated from marginal regions of the leaf common primordium and accompanying this process another key developmental event is the establishment of boundaries between leaflet primordia (Figures 2A,E). The boundary that separates two cell groups of organs has a specific feature of restricted cell growth relative to surrounding tissues, which relies on its unique gene expression profiles and hormone signaling programs (Zadnikova and Simon, 2014; Hepworth and Pautot, 2015). The best-characterized boundary is the domain separating the lateral organs from the SAM, where auxin and brassinosteroids (BRs) are down-regulated. The LATERAL ORGAN BOUNDARY DOMAIN (LBD) family gene LATERAL ORGAN BOUNDARIES (LOB) is expressed in the SAM-to-organ boundary and functions to maintain a low level of BRs, which subsequently restricts cell growth and division. CUP-shaped COTYLEDON (CUC)/NO APICAL MERISTEM (NAM) is a class of plant-specific NAC transcription factors that plays a central role in maintaining growth repression in boundaries (Aida et al., 1997; Blein et al., 2008). In addition to controlling SAM and organ separation, CUC/NAMs are also required for the boundary formation between leaflet primordia during compound leaf development (Blein et al., 2008). The silencing or mutation of *CUC*/*NAM* in different species all resulted in different degrees of fusion and decrease in the number of leaflets, whereas ectopic expression of *CUC/NAM* genes resulted in a compound leaf phenotype of increased leaflet number (Blein et al., 2008; Berger et al., 2009; Wang et al., 2013; Rast-Somssich et al., 2015; Jiao K. Y. et al., 2019). In *M. truncatula*, the *CUC*/*NAM* homologous gene *MtNAM* is also specifically expressed in the boundary region of leaflets (Figure 3C), and mutations lead to leaflet fusion (Cheng et al., 2012). Leaves of the *sgl1 mtnam* double mutant greatly resembled the phenotype of the *sgl1* single mutant with a single leaflet pattern, while *MtNAM* expression is reduced in the *sgl1* mutant but *SGL1* expression was not altered in the *mtnam* mutant (Cheng et al., 2012), indicating that *SGL1* is likely epistatic to *MtNAM* during the trifoliate leaf development and that the leaflet initiation is a prerequisite for leaflet delimitation.

Recently, the class M KNOX proteins, which lack the homeodomain, have been identified in *Arabidopsis thaliana* and tomato (Kimura et al., 2008; Magnani and Hake, 2008). Ectopic expression of the class M *KNOX* gene *KNATM* in *A. thaliana* leads to elongated petiole and narrow blade (Magnani and Hake, 2008). In tomatoes, the gain-of-function of the class M KNOX protein PETROSELINUM (PTS) increased the compound leaf complexity (Kimura et al., 2008). The *M. truncatula Fused Compound Leaf1* (*FCL1*) gene is the orthologous gene of *KNATM*/*PTS* (Peng et al., 2011). Compound leaves in *fcl1* mutants are simplified with fused or clustered leaflets and shortened petioles, but without rachises. Similar to that of *sgl1* mutants, the reduction in the petiole length in the *fcl1* mutants is likely caused by reduced cell division but not cell expansion. During the earliest developmental stages, the *FCL1* expression corresponds to the sites of leaf initiation (P0 to P1); at subsequent developmental stages, *FCL1* was expressed at the proximal domain of developing leaf primordia (Figure 3C); in older developing leaves, *FCL1* was expressed at petiole, rachis and the base of folded blades. This expression pattern of *FCL1* was greatly consistent with its roles in boundary separation and proximal–distal axis development, but the underlying mechanism remains undetermined.

The *M. truncatula WRINKLED FLOWER AND LEAF* (*WFL*) encodes a 3-ketoacyl-CoA synthase involved in the biosynthesis of very-long-chain fatty acids and cuticular wax. The *wfl* mutants have dramatic developmental defects in leaves and floral organs, exhibiting wrinkled leaves throughout the growth period and fused floral organs during the reproductive period (Yang et al., 2021). *WFL* was expressed in epidermal cells of the SAM, leaf primordia, and floral organs. This study is in accordance with the previous report that the functional cuticle is important for maintaining lateral organ separation (Liu et al., 2021).

## Roles of the Leaf Polarity Genes in the Leaf Pattern Formation

Once leaf founder cells are specified at the SAM flank, the leaf polarity simultaneously begins to be established through highly organized cell division and differentiation along three axes: adaxial–abaxial (broadly equivalent to the dorso-ventral), mediolateral, and proximal–distal (Efroni et al., 2010). The side of the leaf primordium facing the SAM is called the adaxial/dorsal face, mainly responsible for capturing sunlight and photosynthesis, and the other side called the abaxial/ventral face is mainly responsible for gas exchange. The region connecting the adaxial and abaxial surfaces is defined as the middle domain. The mediolateral polarity represents the horizontal expansion of the blade. The proximal–distal polarity determines the growth direction of the leaf primordia and the relative placement of the blade and petiole. The proximal portion is close to SAM, which differentiates into petiole, while the region farthest from the SAM is the distal portion where the blade forms. Although the pattern of the leaf polarity is establishment along three axes, the adaxial–abaxial axes play a decisive role. Only when the adaxial–abaxial polarity is established, the leaf can be extended, and the pattern of proximal–distal and the mediolateral can be further established (Efroni et al., 2010; Manuela and Xu, 2020).

### Adaxial–Abaxial Polarity Genes

The MYB domain protein encoded by the *ASYMMETRIC LEAVES1*/*ROUGH SHEATH2*/*PHANTASTICA* (*ARP*) gene plays an important role in leaf initiation and the establishment of leaf adaxial–abaxial polarity (Waites et al., 1998; Timmermans et al., 1999; Byrne et al., 2000; Harrison et al., 2005). Mutations in *PHANTASTICA* of *Antirrhinum* abolished adaxial cell types and inhibited laminar growth (Waites et al., 1998). In *A. thaliana* and *C. hirsuta*, *KNOX I* and *ARP* genes are expressed in mutually exclusive domains, and the maintenance of the repressed state of *KNOX I* genes in the leaf primordium depends on *ARP* genes (Byrne et al., 2000; Hay and Tsiantis, 2006). The antagonism between *ARP* and *KNOX I* is also important for the compound leaf development of *C. hirsuta* (Hay and Tsiantis, 2006; Rast-Somssich et al., 2015). *C. hirsuta chas1* mutants have an altered expression pattern of *KNOX I* genes in leaf primordia, leading to an increased leaflet production. In tomatoes, down-regulation of *ARP* results in a switch from pinnate into peltately palmate compound leaves with abaxialized petioles and reduced leaflet number (Kim et al., 2003). Loss-of-function of the pea *ARP* ortholog *CRISPA* causes various leaf abnormalities, including crisp leaf organs, narrowing leaflets, the curvature of petiole and rachis, and ectopic stipules on its petiole-rachis axis which is also associated with ectopic *KNOX I genes* expression (Tattersall et al., 2005). The *M. truncatula mtphan* mutants also exhibited severe compound leaf defects, including curling and deep serration of leaf margins, shortened petioles, increased rachises, petioles acquiring motor organ characteristics, and ectopic development of petiolules (Ge et al., 2014; Zhou et al., 2014). *MtPHAN* expresses throughout the SAM as well as in adaxial and abaxial sides of developing leaf primordia; *KNOX I* genes appear to uncouple from the *PHAN* regulation. Taken together, the effect of ARPs on leaf development and on *KNOX I* expression varies in different species, but it seems that, in compound leaf development, ARPs have much more important and complex roles on the petiole-rachis identity regulation than what is previously recognized.

The identity of adaxial surface cells depends on the activity of class III homeodomain-leucine zipper (HD-ZIP III) transcription factors, including PHABULOSA (PHB), PHAVOLUTA (PHV), and REVOLUTA (REV) (McConnell et al., 2001; Ochando et al., 2008). Single loss-of-function of *PHB*, *PHV*, or *REV* has no significant effect on the polarity development of lateral organs; simultaneous loss-of-function of *PHB PHV* and *REV* abaxializes cotyledons, abolishes primary apical meristem, and in severe cases, eliminates the bilateral symmetry of lateral organs (Emery et al., 2003). It is well recognized that the adaxial expression of *PHB PHV* and *REV* is restricted by *microRNA165/166* (*miR165/166*), which are expressed in the abaxial domain and mediate the cleavage and degradation of *HD-ZIP III* mRNAs (Rhoades et al., 2002; Tang et al., 2003). Mutations in the homologous gene of *PHB* results in curly leaf in cucumber (*Cucumis sativus*) (Rong et al., 2019); in rice, *LATERAL FLORET 1* (*LF1*) is homologous to Arabidopsis *REV* and the gain-of-function mutant *lf1*, in which *LF1* was expressed ectopically because *miRNA165*/*166* could not act on the mutated *LF1* mRNA, showed a phenotype of highly abaxialized and rolled leaves (Zhang et al., 2017; Zhang et al., 2021). There is only one *PHB*/*PHV* gene but two *REVOLUTA* genes in *M. truncatula* (Zhou et al., 2019). Most leaves in the *mtrev1* mutant consist of five leaflets arranged pinnately, with some cases of a trumpet-shaped terminal leaflet, of which the adaxial side is surrounded by the abaxial side. *MtREV1* was preferentially expressed in the adaxial domain of leaf primordia at different developmental stages, and this polarized expression pattern would also depend on post-transcriptional regulation by *miR165/166*. Overexpression of *MtmiR166*-insensitive *MtREV1* showed ectopic blade tissues forming along the midvein and on the abaxial side of the leaf (Zhou et al., 2019). These suggest that *MtREV1* plays an important role both in maintaining the adaxial polarity of leaves and in the trifoliate pattern formation, and it will be interesting to further investigate how *MtREV1* regulates compound leaf pattern formation and the nature of the relationship between adaxial–abaxial polarity and compound leaf patterning. The YABBY (YAB) family of transcription factors confers abaxial identity in *A. thaliana* (Siegfried et al., 1999), and *MtYAB3* is similarly specific to the abaxial side in *M. truncatula*, while in *mtrev1* mutants, the expression of *MtYAB3* was observed in both adaxial and abaxial sides of leaf primordia (Zhou et al., 2019). However, the remaining *yab* mutants of *M. truncatula* were not characterized and the role of *MtYAB* genes needs to be further investigated.

In addition to *YAB* genes, the above-mentioned ARFs are another type of determinant of abaxial cell fate (Pekker et al., 2005). ARFs are regulated by a group of ta-siRNA named ta-siARFs, which are originated from the cleavage of TAS3 transcripts by ARGONAUTE7 (AGO7) coupled with *miR390* (Adenot et al., 2006; Garcia et al., 2006; Hunter et al., 2006). It has been reported that the *M. truncatula MtAGO7* is required for the biogenesis of ta-siARFs to negatively regulate the expression of *MtARF3* (Zhou et al., 2013). *MtAGO7* is predominantly expressed on the adaxial sides of both the leaf and leaflet primordia and its loss-of-function mutation results in lobed leaf margins and more widely spaced lateral organs. In wild-type, the ta-siARFs assumed to be expressed in the adaxial domain, and expression of *MtARF3* is detected in the abaxial domain; but in *mtago7* mutants, putative ta-siARFs are dramatically reduced and the *MtARF3* was highly expressed and extended into the adaxial domain (Zhou et al., 2013). Plants overexpressed the original MtARF3 exhibited downward-curled leaves and showed more serrations along the leaf margin, whereas overexpression of a mutated ARF3 carrying two altered ta-siARF target sites was phenotyped with obvious lobed leaf margin, mimicking the *ago7-1* phenotype (Zhou et al., 2013).

As mentioned above, a recent study suggested that MtARF3 can directly interact with specific auxin response elements (AuxREs) in the *PALM1* promoter and functions as a repressor to negatively regulate *PALM1* transcription, while *MtARF3* overexpression results in palmate-like pentafoliate leaves with radialized blades, which, to some degree, resembled the phenotype of the *palm1* mutant (Peng et al., 2017). The MtARF3-PALM1 module may benefit from the involvement of *MtPHAN* and *MtAGO7* in the adaxial domain. In either *mtphan* or *mtago7* single mutants, *MtARF3* expression was similarly elevated and detected in both the adaxial and abaxial domains; more surprisingly, in the *mtphan mtago7* double mutants, *MtARF3* transcripts increased drastically and even appeared much higher in the adaxial domain than the abaxial domain, accompanying the downregulated *PALM1* expression and the change of compound leaf pattern from trifoliate to palmate-like pentafoliate with radialized blades. These interactions among MtPHAN, MtARF3, and PALM1 suggest a complex relationship among adaxial–abaxial polarity development, auxin signaling program, and compound leaf patterning.

### Mediolateral Polarity Genes

Formation of the leaf blade requires growth along the mediolateral axis, and *WOX1* genes were found to be specifically involved in this process in diverse eudicot species (Vandenbussche et al., 2009; Nakata et al., 2012; Zhuang et al., 2012; Vandenbussche, 2021; Wang C. et al., 2021). In *A. thaliana*, two *WOX* family genes, *WUS-related HOMEOBOX1* (*WOX1*) and *PRESSED FLOWER* (*PRS*)/*WOX3*, are expressed at the middle domain between the adaxial and the abaxial domains at the P2 stage and the leaf margin at later stages. The WOX1 and PRS/WOX3 can inhibit the expression of adaxial–abaxial characteristic genes in the middle domain, whereas their own gene expression is repressed by *KANADI* (*KAN*) feedback in the abaxial domain (Nakata et al., 2012). In compound leaf development of *M. truncatula*, the *WOX1* ortholog *STENOFOLIA* (*STF*) has conserved roles in promoting lamina outgrowth along the mediolateral axis (Tadege et al., 2011). The *stf* mutants showed a very narrow blade or bladeless phenotype with severe disruption of vein patterning. Similar to the expression patterns of *WOX1* and *PRS/WOX3* in Arabidopsis, *STF* is specifically expressed at the adaxial–abaxial junction of the early leaf primordia, and at the margin of the distal half portion of the leaflets in young developing leaves. The STF protein recruits the TOPLESS (MtTPL) protein to directly repress the LBD family gene *MtAS2* expression and promote cell proliferation at the adaxial–abaxial boundary (Zhang et al., 2014). A recent study found that *MtWOX9* is an abaxial factor required for proper blade outgrowth in *Medicago*, and STF represses *MtWOX9* expression by directly binding to its promoter at multiple sites (Wolabu et al., 2021). It is, therefore, likely that STF establishes and maintains a cell proliferation zone at the adaxial–abaxial junction in the middle mesophyll and leaf margin by keeping adaxial and abaxial polarity factors away from this region (Figure 3D).

### Proximal–Distal Polarity Genes

*Medicago truncatula* trifoliate leaves exemplify global and local polarity along the proximal–distal axis (Figure 2E). The global proximal–distal polarity is manifest in the distribution of distinct specialized organs along the proximal–distal axis: the proximal portion being of a petiole with a pair of stipules located at the proximal end, and the distal portion of three ovate leaflets connected to a central rachis through their pulvini at the lamina base. Each leaflet exhibits independent local proximal–distal polarity which is exemplified by the distal half portion of the lamina forming the acute apex and the proximal half forming the cuneate or slightly convex base, and by the marginal serrations forming in the distal portion (∼75%) but absent in the proximal portion (∼25%).

In *A. thaliana*, two BTB/POZ domain-ankyrin repeat proteins BLADE-ON-PETIOLE1 (BOP1) and BOP2 are reported to regulate proximo-distal patterning and the *bop1 bop2* double mutant develops blade-on-petiole structures (Ha et al., 2007). In pea, *coch* mutants carry mutations in the BOP1 homolog and have stipule phenotypes that vary from weak modifications (asymmetric shape) to spoon-like leaf structures and even complete conversion into complex pea leaf structures (Couzigou et al., 2012). *COCH* gene was expressed at the base of the developing leaf where stipules are formed. The Medicago *BOP1* homolog, *NOOT*/*MtBOP1*, loss-of-function phenotype were simplified stipules with a reduced number of serrations (Couzigou et al., 2012). Recently, the Pea *Stipules reduced* (*St*) gene was identified and it encodes a C2H2 zinc finger transcription factor that is regulated by *COCH* (Moreau et al., 2018). *St* regulates both cell division and cell expansion in the stipule, and it has one highly homologous gene in Medicago, which awaits to be characterized in the future.

Both SGL1 and FCL1 are required for the development of petiole and rachis (Wang et al., 2008; Peng et al., 2011). The petiole was significantly shortened in the *sgl1* leaves and the rachis was absent. In *fcl1* mutants, the petiole was also shorter than the wild-type counterparts and the rachis appears to be shortened or completely absent in different alleles. The average length of petiole epidermal cells was indistinguishable between *fcl1* mutants and wild-type plants, indicating the reduced petiole elongation may be due to altered cell division activity in *fcl1* mutants. Mature leaves of *sgl1 fcl1* double mutants were simple, resembling those of *sgl1* mutants, and besides completely lacked petioles (Peng et al., 2011).

A novel nucleus-localized protein containing a putative Myb/SANT-like DNA-binding domain and a PKc kinase domain, AGAMOUS-LIKE FLOWER (AGLF)/AGAMOUS AND TERMINAL FLOWER (AGTFL), was recently reported to regulate the flower development of *M. truncatula* (Zhao et al., 2019; Zhang et al., 2019; Zhu et al., 2019). Loss-of-function of AGLF results in flowers with stamens and carpel transformed into extra whorls of petals and sepals. The mutants also displayed defects in compound leaf development; both the rachis and petiole were shortened while leaflets clustered together. In *aglf* mutants, leaflet elongation in the longitudinal direction is significantly affected, leading to a heart-shaped lamina with a retuse apex in contrast to the ovate lamina with an acute apex in wild-type plants. The *MtWUS* and *MINI ORGAN1* (*MIO1*)/*SMALL LEAF AND BUSHY1* (*SLB1*) are two important regulators for leaflet elongation along the proximal–distal axis. As mentioned above, the *MtWUS* has conserved functions in the SAM and AM maintenance; besides, *mtwus* mutants produced heart-shaped leaves that showed retuse apex and increased width/length ratio (Meng et al., 2019; Wang et al., 2019). In young leaves, the *MtWUS* was slightly expressed in the leaf lamina as a whole, but it was considered at the tips of the marginal serrations. The *MIO1*/*SLB1* encodes an F-box protein, an ortholog of *A. thaliana* STERILE APETALA (SAP) (Yin et al., 2020; Zhou et al., 2021). The loss-of-function of MIO1/SLB1 severely reduced organ size. Leaves of *mio1* mutants exhibited not only reduced size but also heart-shaped blade morphology. These studies indicate that the lamina growth along the proximal–distal axis of each leaflet is subject to a complex regulation, in which AGLF, MtWUS, and MIO1 would play important roles. However, the detailed mechanism of their action in this developmental process remains to be clarified.

## The Genetic Control of Leaf Organ Size and Marginal Serrations

The overall leaf size is often characterized by its length and width, which are dependent on the growth along the proximal–distal and the mediolateral axes respectively. Cell proliferation and cell expansion are important basic processes promoting growth along the two axes. In *Medicago*, several molecules and signaling pathways responsible for these processes have been recently identified, including phytohormones, transcription factors, and other molecular regulators.

Gibberellin (GA) is involved in various processes of plant growth and development, including leaf expansion, seed germination, induction of flowering, and stem elongation. In *M. truncatula*, *Dwarf and Increased Branching 1* (*DIB1*)/*SMALL AND SERRATED LEAF* (*SSL*) encodes a gibberellin 3β-hydroxylase (GA3ox) enzyme, catalyzing the final step of the biosynthetic pathway for bioactive GAs (Zhang et al., 2020; Wen et al., 2021). The mutant exhibits extreme dwarfism and an increased number of lateral branches, and besides, leaves of the mutant were extremely reduced in all organ sizes and bore a more pronounced leaf margin. Another component of GA biosynthesis in *M. truncatula*, *Mini Plant 1* (*MNP1*), encodes a putative copalyl diphosphate synthase (CPS) implicating in the first biosynthetic step (Guo et al., 2020). The mutant also exhibits extreme dwarfism and very small leaves. MtGA3ox and MtCPS were shown to affect both cell proliferation and elongation because the shortened stem length of two mutant lines is due to a decrease in cell number and size. Three GA 20-oxidases catalyzing the late step of GA biosynthesis, MAIN STEM DWARF1 (MSD1) and its homologs MtGA20ox7 and MtGA20ox8, were recently identified in *M. truncatula*, and they play partially redundant roles in controlling the shoot elongation and the lateral organ size (Li et al., 2021). The *msd1* mutant exhibits a phenotype of the semi-dwarfed main stem but normal leaves, while the *msd1 mtga20ox7 mtga20ox8* triple mutant exhibits a severely dwarf phenotype with markedly reduced leaf size, mimicking the phenotypes of the *dib1* and *mnp1* mutants.

A well-characterized role for brassinosteroids (BRs) is their involvement in cell expansion and organ elongation (Bajguz et al., 2020). BR-deficient or -insensitive mutants of Arabidopsis exhibit phenotypes of dwarf, round leaves, shorter petioles, and infertility. *Brassinosteroids Insensitive 1* (*BRI1*) is required for BR perception and initiation of subsequent signal transduction in Arabidopsis (Li and Chory, 1997). In Medicago, the loss-of-function mutant of *MtBRI1* has typical BR-deficient phenotypes of extreme dwarfness and infertility, and its leaves were thickened, curled, dark green, and greatly reduced in size with the petioles and rachises failed to elongate (Cheng et al., 2017; Kong et al., 2021). *MtDWARF4A* (*MtDWF4A*), a gene encoding a cytochrome P450 protein orthologous to *A. thaliana* DWARF4, is required for the BR biosynthesis. The *mtdwf4a* mutant exhibits mild BR-deficient phenotypes, semi-dwarfism, short petioles, and rachis, but normal fertility (Kong et al., 2021; Zhao et al., 2021). The *MtDWF4A* has a highly homologous copy designated as *MtDWF4B*, it will be critical to explore the possibilities of functional redundancy and diversification between *MtDWF4A* and *MtDWF4B* in the BR biosynthetic pathways (Zhao et al., 2021).

*Medicago truncatula BIG SEEDS1* (*BS1*) encodes a member of group II of the TIFY transcription factor family and it plays a critical role in determining seed and leaf size (Ge et al., 2016). *BS1* is homologous to *A. thaliana PEAPOD1* (*PPD1*) and *PPD2*. Loss-of-function of *BS1* leads to enlarged seeds, fruits, and leaves. As mentioned above, the F-box protein MIO1/SLB1 plays a positive role in organ size determination. Plants overexpressing MIO1/SLB1 had enlarged organs, and this is because MIO1/SLB1 forms part of SKP1/Cullin/F-box (SCF) E3 ubiquitin ligase complex, targeting BS1 proteins for degradation (Yin et al., 2020; Zhou et al., 2021). The pathway of BS1 and MIO1/SLB1 may mainly modulate primary cell proliferation during the early stages of leaf development to control the leaf size.

In *A. thaliana*, CUC2 promotes the establishment of PIN1 convergence points to generate auxin maxima at the tip of serrations (Bilsborough et al., 2011). *M. truncatula* leaflets have serrations on the distal part (∼3/4 midvein) of the lamina margin, and the involved regulators have been recently reviewed (Wang H. et al., 2021). MtPIN10 plays a key role in generating the auxin maxima at the tips of serrations, while MtNAM is also involved in the marginal serration formation (Zhou et al., 2011). *MtLMI1s* encoding HD-Zip I transcription factors homologous to *A. thaliana* LATE MERISTEM IDENTITY1 (LMI1) and *C. hirsuta* REDUCED COMPLEXITY (RCO) (Vlad et al., 2014; Kierzkowski et al., 2019), directly activate the expression of SLM1 to regulate the auxin distribution along leaf margin (Wang X. et al., 2021). The elaboration of leaf margin formation also requires the determination of the degree of marginal indentation, which is regulated by the MtAGO7-mediated TAS3 ta-siRNA pathway through the suppression of MtARF3 expression (Zhou et al., 2013).

## The Pulvinus Development in *Medicago truncatula*

The pulvinus-driven nyctinastic leaf movement is a common and characteristic phenomenon found in legume plants. Many legume plants have compound leaves consisting of multiple leaflets. Each leaflet usually has an independent pulvinus at the base of the lamina, functioning as the motor organ for leaf movement. The determination of pulvinus identity in legumes seemingly shares a conserved genetic network orchestrated by a conserved LBD family gene, namely *SLEEPLESS* (*SLP*) in *Lotus japonicas*, *APULVINIC* (*APU*) in pea (*P. sativum*), and *ELONGATED PETIOLULE1* (*ELP1*)/*PETIOLULE-LIKE PULVINUS* (*PLP*) in *M. truncatula* (Chen et al., 2012; Zhou et al., 2012). Mutations in these genes completely abolished the pulvinus development and leaf movement, while overexpression of *ELP1/PLP* in the *M. truncatula elp1/plp* mutants could not only rescue the pulvinus and movement defect but also lead to highly reduced petioles and rachises, distorted leaf blades, and dwarfed status in some cases, showing a phenotype partially similar to the *mtdwf4a* mutants. Considering that the ELP1/PLP and its orthologs are highly homologous to the Arabidopsis LOB protein which has been shown to directly up-regulates the BR metabolic gene *phyB Activation-tagged Suppressor1-dominant* (*BAS1*), further efforts are needed to determine whether BR accumulation is involved in pulvinus development and what roles *ELP1/PLP* and its orthologs play in this process. The F-box protein MIO1/SLB1 is another factor necessary for robust pulvinus development (Zhou et al., 2021). The pulvini of *mio1/slb1* mutants were shortened even completely absent from the base of its leaflets, leading to a reduced degree of leaflet rotation or a completely impaired leaf movement. Therefore, an interesting and important question is how *ELP1/PLP* and *MIO1/SLB1* control the pulvinus development of *M. truncatula* and whether there is a direct gene-protein relationship between *MIO1/SLB1* and *ELP1/PLP*, which awaits future elucidation.

In addition to the pulvinus playing a central role in leaf movement, other elements of the compound leaf, such as leaflet geometry (the spatial structure and organization of leaflets), would also play important roles, which is proposed recently. In the loss-of-function *mtdwf4a* mutant, the shortened rachises and pulvini in leaves resulted in a physical space constraint among leaflets, leading to their leaflets could not close during the night in contrast to the wild-type (Zhao et al., 2021).

## Conclusion and Perspectives

As shown in the above sections, we now have a rather good knowledge of the genetic networks that control major developmental processes during the pattern formation of *M. truncatula* trifoliate leaves and that correspond to other morphological traits of the leaves. Briefly, one of the most critical developmental processes that determines the trifoliate leaf pattern is the formation of three separated leaflet primordia during early stages, which is characterized by two key events: the initiation of leaflet primordia and the boundary formation between leaflets; and the reported regulatory mechanisms involved in such biological events can be reasonably summarized into at least four aspects: (1) the LFY/SGL1 pathway plays a central role in maintaining the morphogenetic activity of the compound leaf primordia which determines the leaflet initiation from marginal regions of the leaf primordium (Figure 3A); (2) the leaflet initiation and outgrowth is tightly associated with the auxin pathway which is facilitated by multiple components, including MtYUCCA1-catalyzed auxin generation and MtPIN10-driven auxin redistribution (Figure 3B); (3) the boundary formation between leaflets is critically dependent on the function of the boundary-specific *MtNAM/MtCUC2* gene and the *FCL1* gene (Figure 3C); (4) the adaxial–abaxial polarity genes play important roles in determining the compound leaf pattern and there would be a complex crosstalk between the LFY/SGL1 pathway and the regulation of the adaxial–abaxial polarity (Figure 3D).

For a more comprehensive understanding of the molecular mechanism underlying the compound leaf pattern formation of *M. truncatula*, elucidation of the additional key players as well as of the coupled upstream and downstream pathways are required. Several important questions should be addressed in future studies such as the following. At first, the initiation of leaflet primordia and the boundary delimitation between them during pattern formation are two interconnected processes, and it will be important to investigate how they are coordinatively controlled. Secondly, *FCL1* may be involved in regulating NAM/CUC transcription factor-dependent and/or auxin-dependent regulatory networks, and future investigation will be aimed at elucidating the mechanism of FCL1 controlling the leaf pattern formation. Thirdly, the adaxial–abaxial polarity genes *MtREV1*, *MtPHAN*, *MtAGO7*, and *MtARF3* regulate both adaxial–abaxial identity and compound leaf patterning, and meanwhile, they were involved in the auxin signaling pathway, besides, *PINNA1* shows an adaxial-specific expression pattern and is involved in the LFY/SGL1 pathway; therefore, complex links seem to exist between the establishment of adaxial–abaxial polarity, the auxin signaling pathway, and the LFY/SGL1 pathway during compound leaf patterning, and it would be promising to study such links at a deeper molecular level.

The genome sequences are now available for alfalfa, and CRISPR has been shown to efficiently work in it, which should greatly facilitate the translation of basic knowledge obtained in the *M. truncatula* to the future breeding of leaf traits in alfalfa (Chen et al., 2020; Shen et al., 2020). Most importantly, the loss-of-function mutant of *MsPALM1* generated by CRISPR/Cas9 developed palmate-like pentafoliate leaves and besides, the mutated alleles and phenotypes can be stably transmitted to progenies by cross-pollination between two mutants in a transgene-free manner, which may help to accelerate the breeding speed.

## Author Contributions

XM wrote the first draft. LH and JC contributed to wrote and revised the manuscript. LH designed the figures. YL, DW, and BZ provided help and advice, and corrected the manuscript. All authors contributed to the article and approved the submitted version.

## Conflict of Interest

The authors declare that the research was conducted in the absence of any commercial or financial relationships that could be construed as a potential conflict of interest.

## Publisher’s Note

All claims expressed in this article are solely those of the authors and do not necessarily represent those of their affiliated organizations, or those of the publisher, the editors and the reviewers. Any product that may be evaluated in this article, or claim that may be made by its manufacturer, is not guaranteed or endorsed by the publisher.
